# Rapid Identification of Pathogenic Variants in Two Cases of Charcot-Marie-Tooth Disease by Gene-Panel Sequencing

**DOI:** 10.3390/ijms18040770

**Published:** 2017-04-05

**Authors:** Chi-Chun Ho, Shuk-Mui Tai, Edmond Chi-Nam Lee, Timothy Shin-Heng Mak, Timothy Kam-Tim Liu, Victor Wai-Lun Tang, Wing-Tat Poon

**Affiliations:** 1 Department of Clinical Pathology, Pamela Youde Nethersole Eastern Hospital, Chai Wan, Hong Kong, China; hcc604@ha.org.hk (C.-C.H.); tangwlv@ha.org.hk (V.W.-L.T.); 2 Department of Paediatrics & Adolescent Medicine, Pamela Youde Nethersole Eastern Hospital, Chai Wan, Hong Kong, China; taism1@ha.org.hk (S.-M.T.); liukt1@ha.org.hk (T.K.-T.L.); 3 Department of Medicine, Pamela Youde Nethersole Eastern Hospital, Chai Wan, Hong Kong, China; leecn2@ha.org.hk; 4 Centre for Genomic Sciences, Li Ka Shing Faculty of Medicine, The University of Hong Kong, Pokfulam, Hong Kong, China; timothy.mak@hku.hk

**Keywords:** Charcot-Marie-Tooth disease (CMT), hereditary motor and sensory neuropathy (HMSN), pathogenic variants, gene panel, next-generation sequencing (NGS)

## Abstract

Charcot-Marie-Tooth disease (CMT) is a common inherited peripheral neuropathy affecting up to 1 in 1214 of the general population with more than 60 nuclear genes implicated in its pathogenesis. Traditional molecular diagnostic pathways based on relative prevalence and clinical phenotyping are limited by long turnaround time, population-specific prevalence of causative variants and inability to assess multiple co-existing variants. In this study, a CMT gene panel comprising 27 genes was used to uncover the pathogenic mutations in two index patients. The first patient is a 15-year-old boy, born of consanguineous parents, who has had frequent trips and falls since infancy, and was later found to have inverted champagne bottle appearance of bilateral legs and foot drop. His elder sister is similarly affected. The second patient is a 37-year-old woman referred for pre-pregnancy genetic diagnosis. During early adulthood, she developed progressive lower limb weakness, difficulties in tip-toe walking and thinning of calf muscles. Both patients are clinically compatible with CMT, have undergone multiple genetic testings and have not previously received a definitive genetic diagnosis. Patients 1 and 2 were found to have pathogenic homozygous *HSPB1*:NM_001540:c.250G>A (p.G84R) variant and heterozygous *GDAP1*:NM_018972:c.358C>T (p.R120W) variant, respectively. Advantages and limitations of the current approach are discussed.

## 1. Introduction

Charcot-Marie-Tooth disease (CMT) is the most common inherited peripheral neuropathy estimated to affect 1 in 1214 of the general population [1]. Over 60 nuclear genes have been implicated in the pathogenesis of this group of disorders, and the genetic cause of about 50% of clinically diagnosed CMT remains unidentified [2]. The inheritance of this group of disorders is variable, and cases with mutations that are autosomal dominant [3,4], autosomal recessive [5,6], X-linked dominant [7], X-linked recessive [8] and de novo [9] have all been described. Even within the same causative gene, CMT inheritance has been reported to vary in a site-specific manner [10]. Furthermore, clinical severity and prognosis of CMT also depends on the presence of modifying mutations [11]. Identification of the exact or most probable underlying genetic mutation is therefore of importance in the clinical management, genetic and reproductive counselling of patients diagnosed to have CMT.

In diseases with multiple causative genes such as CMT, diagnostic algorithms based on clinical phenotyping and known prevalence of genetic defect has been preferred [12]. While large-scale genotyping studies had elucidated more detailed genetic epidemiology of CMT and led to continuous refinement of such algorithms [13], the concurrent advancement of genomic technologies has more recently allowed the simultaneous interrogation of multiple gene targets with comparable cost-effectiveness [14,15,16]. The advantage of this alternative approach is multifold: first, there is potential cost-savings in labour, reagents and possibly a reduction in total turnaround time in the diagnostic laboratory; second, it allows a more confident, broad-based genetic diagnosis to be established as known disease genes are tested with less a priori assumptions regarding the prevalence of specific genetic mutations and genetic aetiology of the disease; third, and perhaps most importantly, it provides information for elucidating the roles of co-existing, disease-modifying and cumulative pathogenic effect of multiple variants [17].

In this study, a commercial CMT gene panel comprising 27 known disease-causing genes (Table 1) was used to screen for mutations in autosomal dominant, autosomal recessive, X-linked forms of CMT, and hereditary motor and sensory neuropathy. Next-generation sequencing with high coverage (≥100×) was used for detection of SNPs (single nucleotide polymorphisms) and indel variants for two clinically diagnosed CMT patients who had previously undergone conventional, step-wise genetic testing but have not received a positive genetic diagnosis. Gene-panel sequencing findings were validated and interpreted in the context of family and clinical history. Finally, iterative *in silico* simulation was used to suggest a clinically useful range of sequencing coverage for detecting CMT mutations by gene-panel sequencing experiments in the clinical laboratory.

## 2. Results

### 2.1. Case Report

#### 2.1.1. Case 1

**Figure 1:**
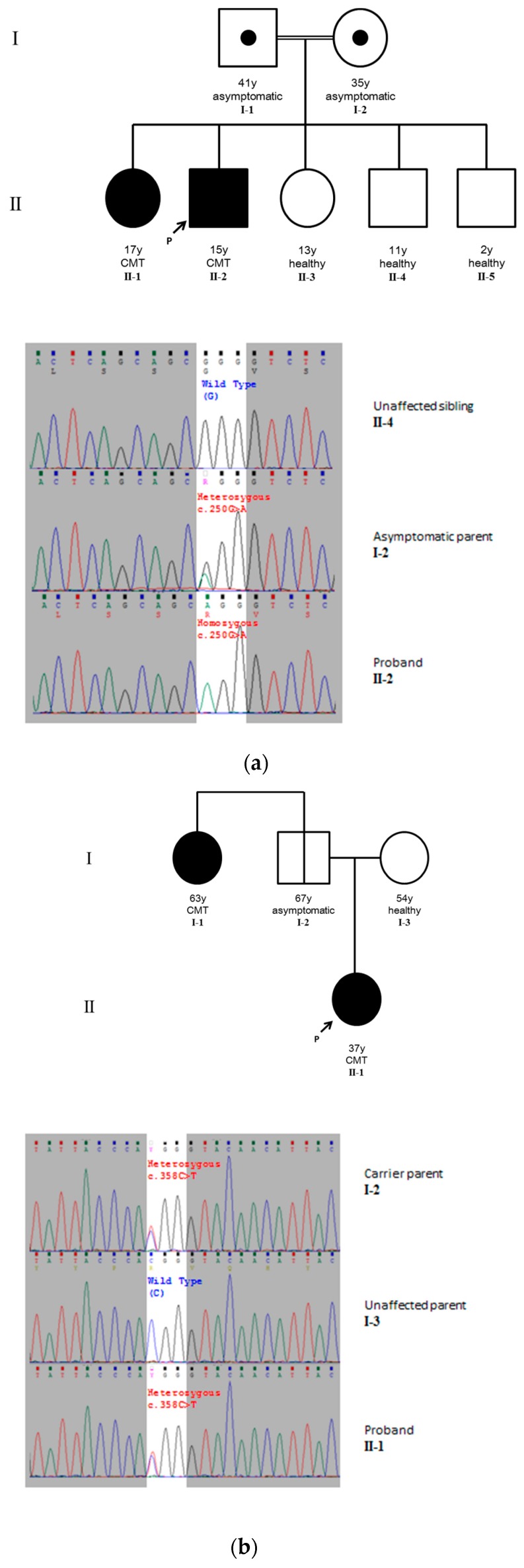
(**a**) Pedigree showing the autosomal recessive inheritance in Case 1 with selected electrophoretograms from Sanger sequencing in the cascade screening. Unaffected siblings II-3 to II-5 are all negative for the *HSPB1*:NM_001540:c.250G>A (p.G84R) variant. Both parents (I-1 and I-2) are heterozygous for the variant, whereas the proband (II-2) and his affected elder sister (II-1) are both homozygous for the pathogenic variant; (**b**) Pedigree showing the autosomal dominant inheritance in Case 2. The proband (II-1), her father (I-2) and her paternal aunt (I-1) are all heterozygous for the pathogenic variant *GDAP1*:NM_018972:c.358C>T (p.R120W), and the proband’s mother (I-3) is homozygous for the wildtype allele. The phenotype of I-2 may be explained by the incomplete penetrance of dominant *GDAP1* mutations, which is discussed in text. In the figure, filled circles and squares denote affected females and males, respectively. A central dot in a symbol denotes an asymptomatic carrier and a line through the symbol denotes a currently asymptomatic carrier who may later develop the disease. The probands are marked with an arrow and a letter “p”.

Patient 1 (**II-2**, Figure 1a) is a 15-year-old boy who tripped and fell frequently since infancy. He fractured his ankle at 10 years old and started to walk with an awkward gait after his plaster was removed. Subsequently, he developed slowly progressive weakness of his lower limbs.

Examination revealed muscle wasting of both distal leg and foot muscles, with inverted champagne bottle appearance of bilateral legs and foot drop. He walked with a high stepping gait. Examination of his upper limbs showed mild thenar and hypothenar muscle wasting with normal power. The peripheral reflexes and sensation were normal. He had no facial weakness, ptosis or respiratory problem. Nerve conduction studies (NCS) showed absent compound muscle action potential upon supra-maximal stimulation of both peroneal and tibial nerves. The nerve conduction velocities of the median and ulnar nerves and the sensory nerve action potential of both sural nerves were normal. The result was suggestive of axonal degenerative motor neuropathy. Previous genetic tests showed no duplication of *PMP22* or mutation in *GJB1* and *MFN2*.

The patient’s parents are cousins, and there is also a history of consanguineous marriage in the last three generations of the family. Upon inquiry, the 17-year-old sister of Patient 1 was noted to be clumsy since infancy and could only walk independently from 18 months onwards. She developed frequent falls since 14 years of age but she remained ambulatory. Examination revealed muscle wasting of distal lower limb muscles and bilateral foot drop. She also walked with a high stepping gait. Nerve conduction studies also revealed similar findings to that in Patient 1. No previous genetic test was performed in this sibling.

#### 2.1.2. Case 2

Patient 2 (**II-1**, Figure 1b) was referred to us for pre-pregnancy genetic diagnosis and counselling at the age of 37. She enjoyed good past health until the age of 18, when she was noticed to have some difficulties in walking. During her twenties, the weakness progressed further and she developed difficulties in tip-toe walking and running. Her family member also noticed thinning of her calves. There was no upper limb or bulbar involvement. One of her paternal aunts developed similar walking problems at the age of 45. The patient’s parents are asymptomatic.

On examination, there was muscle wasting over the anterior and posterior compartment of bilateral lower limbs. Hammer toe and pes cavus were noted. The lower limb reflexes were absent despite reinforcement. There was decrease in distal muscle power over bilateral ankles to grade four minus (Medical Research Council (MRC) scale) while the proximal muscle power of her lower limbs was largely preserved. There was no cerebellar sign. Gait examination revealed a high-stepping gait and the patient was unable to perform tip-toe walking. The upper limb examinations were normal. NCS showed a mild decrease in amplitude in bilateral peroneal nerves. NCS on Patient 2’s mother only showed diabetes-related nerve conduction changes; and that performed on her father was essentially normal. NCS on her clinically affected paternal aunt was compatible with axonal type motor and sensory neuropathies over bilateral upper and lower limbs. Previous genetic tests of the proband showed no duplication of *PMP22*.

### 2.2. Identification of Pathogenic Variants

#### 2.2.1. Case 1

A total of 204 variants, comprising 162 SNPs and 42 indels, were called by HaplotypeCaller with confidence score ≥50. Using wANNOVAR, after filtering by allele frequency to exclude variants with allele frequency ≥0.05 in the 1000 Genome project and Exome Aggregation Consortium (ExAC) database, three exonic or splice-site variants remained. MutationTaster identified 209 analysable alterations (some variants mapped to more than one transcript) and three variants were predicted to be disease-causing. Only one variant was predicted to be Mendelian disease-causing by KGGSeq (Table 2).

Among the shortlisted variants, only the *HSPB1* non-synonymous SNP variant has a homozygous genotype and is compatible with the autosomal recessive inheritance demonstrated in the pedigree of the patient; database search with the Human Gene Mutation Database (www.hgmd.cf.ac.uk) revealed the known mutation CM084860, which has been described in an asymmetrical late onset form of CMT [10] and demonstrated to cause decreased HSPB1-HSPB6 heterooligomer formation, hence decreased chaperone activity [18]. The homozygous pathogenic variant in the *HSPB1* gene was confirmed in the patient by bi-directional Sanger sequencing. Cascade testing confirmed the homozygous *HSPB1*:NM_001540:c.250G>A (p.G84R) variant in the patient’s symptomatic elder sister and both parents were found to be heterozygous for the variant; the patient’s three other siblings did not carry the mutation (Figure 1a).

#### 2.2.2. Case 2

A total of 193 variants, comprising 160 SNPs and 33 indels, were called by HaplotypeCaller with confidence score ≥50. Using wANNOVAR with similar filtering criteria as Case 1, four variants remained. Analysis with MutationTaster identified 199 analysable alterations, and three variants were predicted to be disease-causing. Again, only one variant was predicted to be Mendelian disease-causing by KGGSeq (Table 2).

The heterozygous *GDAP1*:NM_018972:c.358C>T (p.R120W) variant was initially reported as a mutation in autosomal recessive form of CMT [19], and was later found also in the autosomal dominant form of the disease [20]. It is also listed in the Human Gene Mutation Database as known mutation CM032927. The mutation was successfully confirmed in the patient by bi-directional Sanger sequencing. On cascade screening, the patient’s father (clinically asymptomatic, no definite electrophysiological features of CMT) and her paternal aunt (affected, compatible electrophysiological changes over bilateral upper and lower limbs) were both found to harbour the pathogenic *GDAP1* variant. The variant was not detected in the *GDAP1* gene of the patient’s mother (Figure 1b). These findings are compatible with the previously reported incomplete penetrance of the *GDAP1* p.R120W substitution in an Ashkenazi Jew family [21].

### 2.3. Estimation of Minimal Sequencing Depth for Genetic Diagnosis

**Figure 2:**
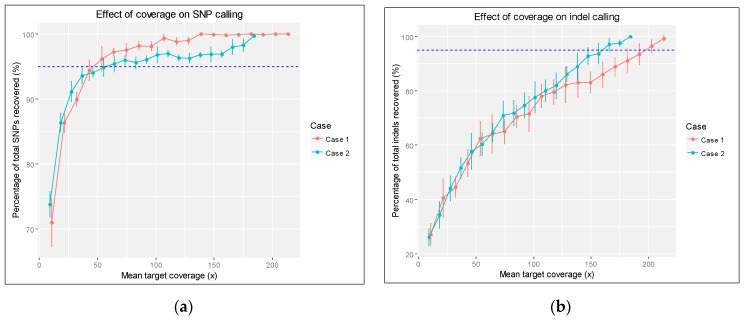
Effect of sequencing coverage on (**a**) SNP and (**b**) indel calling. Error bars represent standard deviation of percentage of SNP and indel recovered at each level of coverage, calculated from 10 replicates. The dashed line (in blue) denotes an arbitrary 95% threshold of the total number of variants recovered, using the variant calls from the full sequencing data as the pseudo-truth set.

With mean target coverage of 213.4× for Case 1 (target coverage ≥2×: 99.09%, ≥10×: 98.64%, ≥50×: 90.98%) and 184.2× for Case 2 (target coverage ≥2×: 99.29%, ≥10×: 98.85%, ≥50×: 89.50%), random sampling of reads was performed from 100% of reads with 5% decremental steps down to 5%, with 10 samples at each level of coverage. Using original data as the pseudo-truth set, with decreasing depth of coverage, the mean proportion of SNPs called decreased from 100% (162.0 ± 0.0) at full coverage to 71.0% (115.0 ± 6.0) at 5% (10.7×) coverage for Case 1 and 99.7% (159.5 ± 0.5) to 73.8% (118.0 ± 3.2) for Case 2 (Figure 2a).

Similarly, the mean proportion of indels called decreased from 99.3% (41.7 ± 0.5) to 27.1% (11.4 ± 1.7) for Patient 1 and 100% (33.0 ± 0.0) to 26.1% (8.6 ± 1.1) (Figure 2b). Thus, the average coverage required to correctly call 95% of the SNPs (C_snp95_) was determined to be 43× < C_snp95_ < 53× for Case 1 and 55× < C_snp95_ < 64× for Case 2. The average coverage required to correctly call 95% of the indels (C_indel95_) was 192× < C_indel95_ < 203× for Patient 1 and 156× < C_indel95_ < 166× for Patient 2.

The minimal mean target coverage at which the homozygous variant *HSPB1*:NM_001540:c.250G>A from Patient 1 could be called from all 10 iterations was 21.3× (10% of original coverage, average coverage of 5.4× at the SNP site), and that of the heterozygous variant *GDAP1*:NM_018972:c.358C>T from Patient 2 was 9.2× (5% of original coverage, average coverage of 16.4× at the SNP site).

## 3. Discussion

In this study, we report the successful application of targeted gene-panel sequencing in the genetic diagnosis of two cases of CMT. Because of the large number of genes implicated in this heterogeneous group of disease, this and other studies [22,23,24,25] together highlight the practical advantage of high-throughput sequencing in rapidly reaching the specific genetic diagnosis over previously proposed stepwise diagnostic algorithms [26,27,28]. As illustrated in Case 1, the patient had undergone screening for *PMP22* duplication, mutation of *GJB1* and *MFN2* and active investigation for more than two years before the gene-panel method was attempted. It is perhaps less surprising if the logistics, quality control and accreditation requirements peculiar to a clinical laboratory are considered: the turn-around time of testing even a single gene is usually more than a few weeks even in resource-rich countries (https://ghr.nlm.nih.gov/primer/testing/costresults). This is obviously in stark contrast to many research centres where researchers using flexible workflows can quickly produce results.

Patients having disease mutations in genes at the distal end of the diagnostic algorithm often have delayed genetic diagnosis. While the sequential testing of disease genes ranked by prevalence and clinical picture may result in certain “cost-savings”, it can also mean an unacceptably long time-to-diagnosis on an individual and community level. In Case 2, the rapid screening role of the gene panel is well-illustrated: contemplating pregnancy at age of 37, the patient faces an age-dependent increase in adverse foetal and maternal outcome [29]. Recent studies suggest that a short waiting time—which is often facilitated by faster test turn-around time—is among the more important factors to patients attending clinical genetic services [30] and the preference for pre-implantation genetic diagnosis decreases with a long waiting list even for couples with a high risk of transmitting genetic disorders to their offspring [31].

While the genetic diagnoses in both cases were successful and relatively straightforward, from the perspective of a clinical laboratory, we also point out certain limitations and caveats in the application of the current approach. First, high-quality sequence of adequate coverage is essential for the successful application of this workflow, yet current technology still mandates a trade-off between breadth and depth of sequencing. While exome or whole genome data may be acquired and restricted variant calling be performed to flexibly simulate the effect of a gene panel and extend analysis into deep non-coding regions as required, the use of a physical gene panel, either by nucleic acid capture or target amplification, still provides a higher and potentially more even coverage of the target regions [32]. Statistical or rule-based variant filtering [33,34,35] and downstream Sanger sequencing validation may be required unless variant calling is highly confident. As seen from the down-sampling experiment, although the original coverage achieved is more than two times the 43× to 64× coverage estimated to achieve 95% of the SNP-calling sensitivity, the coverage margin, especially for indel-calling, can certainly be improved (Figure 2b). It is also possible that further improvement in sequencing chemistry, coverage depth and variant-calling algorithms may further reduce the number of “confident” yet spurious variant calls and decrease the workload for manual validation (Table 2; also see supplementary material II). Additionally, we note that the rarefaction curves presented thereof are optimistic estimates, since the pseudo-truth set is inherently biased towards SNPs and indels that are more easily (hence already) called at low coverages using existing pipelines; as such, no attempt was made to calculate the asymptotic values of total number of SNPs and indels to avoid misleading conclusions. An alternate approach to reduce this bias would be the cross-validation with alternate variant detection methods, such as SNP arrays [14], at different levels of sequencing coverage.

Second, and perhaps more importantly, the genomic alteration leading to the disease that can be detected by this approach is limited to single-nucleotide variants and small insertions and deletions. Determination of large insertions and deletions, copy number variations and genomic rearrangements, even with state-of-the-art analytical software tools [36], is still met with much difficulty especially in gene-panel assays [37]. As gene duplications and deletions are important mechanisms implicated in the pathogenesis of CMT, over-reliance on this single modality of molecular diagnostics can lead to false-negative results.

Preliminary testing for *PMP22* (chromosome 17p) duplication had been performed in both Case 1 and Case 2. Certainly, it may be argued that, due to the prevalence of *PMP22* duplication and deletions, particularly in the more prevalent Type 1 (demyelinating) CMT [2], the preferential testing of such instead of direct gene-panel screening may be a more practical approach [13]. However, this is complicated by uncertainties in electrophysiological and clinical phenotyping of CMT. Electrophysiological phenotyping method using nerve conduction velocity [38] has been compromised not only by significant variability within patient groups with the same mutation [39], but also the increased recognition of a group of mutations leading to intermediate nerve conduction velocities [40]. As far as clinical phenotyping is concerned, genetically informed analysis of patient cohort comprising various CMT subtypes also showed marked phenotypic variability within type 1 and type 2 CMT [41]. It is becoming clear that the underlying mutation, instead of electrophysiological, phenotypic or mode of inheritance, characterize the CMT subtypes [2].

For the two cases described in this study, it is uncertain whether the previous genetic test sequence would have been altered by the clinical information and electrophysiological tests: despite nerve conduction velocity findings compatible with type 2 CMT, *PMP22* genetic testing, which should have higher diagnostic yield in type 1 CMT [42], was still performed. In view of such, the early application of gene-panel sequencing in screening for pathogenic variants, in localities where such technologies and interpretive expertise are more easily available, could therefore eliminate unnecessary repeated clinical examinations, electrophysiological studies and surgical biopsies. However, multiplex or massively parallel sequencing, even if only limited to a panel of known disease genes, has a significant risk of producing equivocal results, i.e., multiple variants “of unknown significance” [43], especially when the disease-causing variant is not obvious from initial database search. Clinical correlations and further investigations, therefore, shall be the logical response to the multitude of hypotheses put forth by the clinical sequencing experiment.

## 4. Materials and Methods

### 4.1. Patients and Human Ethics

Seven members of a family of Pakistan descent (Case 1) and four members of a Han Chinese family (Case 2) were examined. Peripheral blood was collected from all 11 of them and gene-panel sequencing was performed for the two probands. The study was reviewed and approved by the Hong Kong Hospital Authority/Hong Kong East Cluster Institutional Review Board Ethics Committee (HKEC-2016-047). Written informed consent for genetic testing and participating in the study was obtained from all subjects or their parents/guardians.

### 4.2. Gene-Panel and Next-Generation Sequencing

Following genomic DNA extraction from peripheral blood, DNA from targeted regions was enriched using SeqCap EZ Choice Library (Roche NimbleGen, Madison, WI, USA) with the custom oligonucleotide library generated against coding exons of 27 CMT disease-related genes (Table 1). The target-enriched DNA was sequenced by the Illumina HiSeq 2000 sequencing platform (Illumina, San Diego, CA, USA). The gene-panel capture and sequencing was performed at the BGI Diagnostics sequencing facilities (BGI, Shenzhen, China).

### 4.3. Variant Discovery and Annotation

The paired-end 100 base-pair (bp) reads were mapped onto the UCSC human reference genome (version hg19) using BWA-MEM (version 0.7.12) [44]. Duplicate reads were removed using Picard Tools (version 1.94, available from https://github.com/broadinstitute/picard/). After realignment around indels and quality score recalibration, variant calling was performed using HaplotypeCaller (minimum Phred-scaled confidence score = 50; limiting to exons listed in Table 1 and their respective 100 bp upstream and 100 bp downstream regions) [45]. Variant quality score recalibration and hard filtering were not performed because of the relatively small dataset and the need to maximize sensitivity in this clinical study. The variant-calling pipeline has been validated using the NA12878 TruSeq Exome enrichment HiSeq 2500 reads (data available from https://basespace.illumina.com/analyses/36605989?projectId=25504495) against the USA National Institute of Standards and Technology (NIST) Genome in a Bottle v3.2.2 (hg19) using Variant Calling Assessment Tool (v3.0) and achieved a single nucleotide variant (SNV) recall and precision of 96.57% and 99.11%; and indel recall and precision of 89.12% and 92.64% (supplementary material I).

All variants called by the pipeline were subjected to wANNOVAR [46], KGGSeq [47] and MutationTaster [48] analysis as follows. Variants were processed using wANNOVAR for functional annotation and to exclude variants not within the exome regions included by wANNOVAR and those with allele frequency ≥0.05 in 1000 Genome project or ExAC database [46]. Pathogenicity prediction of nonsynonymous variants was performed using KGGSeq with default quality control criteria (--gty-qual 20 --gty-dp 4), additional option to predict Mendelian disease-causing and complex disease-causing variants (--mendel-causing-predict best --db-score dbnsfp --db-gene refgene) and reporting variants with allele frequency <0.05 in 1000 Genome project and ExAC database (--db-filter 1kg201305,dbsnp138,exac --rare-allele-freq 0.05) [47]. Additionally, the variants were submitted to MutationTaster for extended pathogenicity detection for synonymous, splice-site and non-coding region variants [48]. The pathogenic variants shortlisted by MutationTaster were cross-checked with wANNOVAR exome region annotation and had their reads-mapping inspected manually in Integrative Genomics Viewer to note unusual patterns of mapping which may indicate sequencing or mapping errors.

### 4.4. Pathogenic Variant Validation and Cascade Screening

Bi-directional Sanger sequencing was performed for pathogenic variants identified to validate and genotype variants, similar to previously described [49]. Briefly, DNA from peripheral blood was extracted using Qiagen QIAamp^®^ DNA Blood Mini Kit (Qiagen, Hilden, Germany) following the manufacturer’s instructions and eluted in 100 μL of Tris-EDTA buffer. Target regions were amplified from extracted genomic DNA by PCR, each 25 μL reaction contains: 12.5 µL AmpliTaq Gold^®^ 360 Master Mix (Applied Biosystems, CA, USA), 1.0 μL 360 GC Enhancer, 25 μM of each of forward and reverse primers, 20 ng purified genomic DNA and 7.5 μL of PCR-grade water. Amplification was performed using a stepdown PCR protocol as follows: initial denaturation at 95 °C for 10 min, subsequent denaturation at 95 °C for 30 s, annealing for 30 s with 1 °C decrement per cycle, from 66 to 56 °C for 10 cycles, followed by 30 cycles at 60 °C for, extension at 72 °C for 1 min, followed by final extension at 72 °C for 10 min. The PCR products were electrophoresed in 2% agarose in 1× TBE buffer electrophoresis, stained with GelStar (Lonza, Basel, Switzerland). Post-PCR clean-up was performed using EXO-SAP IT^®^ (Affymetrix, CA, USA) according to the manufacturer’s protocol. Sanger sequencing was performed using the BigDye Terminator v1.1 Cycle Sequencing Kit (Applied Biosystems, CA, USA) and an ABI 3500 genetic analyzer. Cascade screening for the family members of the affected probands for the pathogenic mutations identified was similarly performed. For Case 1, both parents and four siblings of the proband (including one symptomatic sibling) were screened (Figure 1a). For Case 2, both parents and a symptomatic paternal aunt were screened (Figure 1b). The grandparents and other relatives of both probands could not be reached for the cascade screening.

### 4.5. Down-Sampling Experiment

Rarefaction curves (Figure 2) were used to explore the effect of sequencing depth on variant discovery. To estimate the sequencing coverage needed for discovering clinically significant homozygous and heterozygous variants, average depth of coverage of the target genomic regions was first calculated using CollectHsMetrics command in Picard Tools and a Python script was written to repeatedly execute HaplotypeCaller with -dfrac values from 1.0 (100% of sequencing reads) with decrement steps of 0.05, down to 0.05 (5% of sequencing reads). The number of SNP and indel variants called were plotted against the average coverage with respective standard deviation (*n* = 10).

## Figures and Tables

**Table 1:** Charcot-Marie-Tooth disease (CMT) 27-gene panel used in the current study.

Gene	CMT Phenotype	Inheritance	Chromosome Location
*KIF1B*	CMT 2A1	AD	1p36.22
*MFN2*	CMT 2A2A & 2A2B	AD, AR	1p36.22
*YARS*	Dominant intermediate CMT (DI-CMT) type C	AD	1p35.1
*LMNA*	CMT 2B1	AR	1q22
*MPZ*	DI-CMT type D, CMT 1B, 2I & 2J	AD	1q23.3
*RAB7A*	CMT 2B	AD	3q21.3
*SH3TC2*	CMT 4C	AR	5q32
*FIG4*	CMT 4J	AR	6q21
*GARS*	CMT 2D	AD	7p14.3
*HSPB1*	CMT 2F	AD	7q11.23
*NEFL*	CMT 1F & 2E	AD, AR	8p21.2
*GDAP1*	Recessive intermediate CMT type A, CMT 2K & 4A	AD, AR	8p21.11
*NDRG1*	CMT 4D	AR	8q24.22
*EGR2*	CMT 1D	AD	10q21.3
*SBF2*	CMT 4B2	AR	11p15.4
*MTMR2*	CMT 4B1	AR	11q21
*FGD4*	CMT 4H	AR	12p11.21
*TRPV4*	Hereditary motor and sensory neuropathy (HMSN) IIc	AD	12q24.11
*HSPB8*	CMT 2L	AD	12q24.23
*LITAF*	CMT 1C	AD	16p13.13
*AARS*	CMT 2N	AD	16q22.1
*PMP22*	CMT 1A & 1E	AD	17p12
*DNM2*	DI-CMT type B, CMT 2M	AD	19p13.2
*PRX*	CMT 4F	AR	19q13.2
*MED25*	CMT 2B2	AR	19q13.33
*GJB1*	X-linked dominant CMT type 1	XLD	Xq13.1
*PRPS1*	X-lined recessive CMT type 5	XLR	Xq22.3

**Table 2:** Exonic and splice-site variants shortlisted using wANNOVAR and analysed by MutationTaster and KGGSeq.

Case	Variant	Gene (Variant Type)	wANNOVAR (Exome Aggregation Consortium (ExAC) Overall Minor Allele Frequency (MAF))	MutationTaster Prediction (Prediction Probability, P_correct_)	KGGSeq Prediction (Disease-Casual Probability, P_disease_)
1	chr1:156109095_156109095delA *LMNA*:NM_170707:cDNA.2405_2405delA	Lamin A/C (Heterozygous 3′ UTR indel in a poly-A stretch) *	(excluded) ^1^	Disease-causing (P_correct_ > 0.999)	No prediction ^2^
	chr7:75932279G>A *HSPB1*:NM_001540:c.G250A (p.G84R)	Heat shock protein family B member 1 (Homozygous nonsynonymous SNP)	Shortlisted (no ExAC data)	Disease-causing (P_correct_ > 0.999)	Disease-causing (P_disease_ = 0.681)
	chr11:9861208G>C *SBF2*:NM_030962:c.C3292G (p.L1098V)	SET binding factor 2 (Heterozygous nonsynonymous SNP)	Shortlisted (MAF = 0.0209)	Polymorphism (P_correct_ = 0.054) ^3^	Non-disease-causing (P_disease_ = 3.46 × 10^−^^4^)
	chr11:95595177A>G *MTMR2*:NM_016156: c.T447C (p.Y149Y)	Myotubularin related protein 2 (Heterozygous synonymous SNP)	Shortlisted (MAF = 2.527 × 10^−^^5^, all from South Asian data in ExAC)	Disease-causing (P_correct_ = 1)	No prediction ^2^
2	chr1:10342522G>A *KIF1B*:NM_015074:c.G1227A (p.T409T)	Kinesin family member 1B (Heterozygous synonymous SNP)	Shortlisted (MAF = 0.0328)	Polymorphism (P_correct_ = 2.98 × 10^−^^17^) ^3^	(filtered) ^4^
	chr1:10397567A>G *KIF1B*:NM_015074:c.A3260G (p.Y1087C)	Kinesin family member 1B (Heterozygous synonymous SNP)	Shortlisted (MAF = 0.0325)	Polymorphism (P_correct_ = 5.41 × 10^−^^11^) ^3^	Non-disease-causing (P_disease_ = 0.039)
	chr1:156109095_156109095delA *LMNA*: NM_170707:cDNA.2405_2405delA	Lamin A/C (Heterozygous 3′ UTR indel in a poly-A stretch) *	(excluded) ^1^	Disease-causing (P_correct_ > 0.999)	No prediction ^2^
	chr8:75272419C>T *GDAP1*:NM_018972:c.C358T (p.R120W)	Ganglioside-induced differentiation-associated protein 1 (Heterozygous nonsynonymous SNP)	Shortlisted (no ExAC data)	Disease-causing (P_correct_ > 0.999)	Disease-causing (P_disease_ = 0.500)
	chr11:9990017G>A *SBF2*:NM_030962:c.C1471T (p.L491F)	SET binding factor 2 (Heterozygous nonsynonymous SNP)	Shortlisted (no ExAC data)	Disease-causing (P_correct_ > 0.999)	Non-disease-causing (P_disease_ = 0.044)

^1^ The shortlisting by allele frequency was limited to “exome summary results” per wANNOVAR settings; ^2^ KGGSeq pathogenicity prediction for non-synonymous variants is based on a logistic regression model combining scores from 14 prediction algorithms/models including SIFT (http://sift.jcvi.org/), PolyPhen2 (http://genetics.bwh.harvard.edu/pph2/) and CADD (http://cadd.gs.washington.edu/), thus no prediction is provided for non-coding variants; ^3^ From MutationTaster documentation (http://doro.charite.de/MutationTaster/info/documentation.html), a prediction probability below 0.5 indicates that the Bayesian classifier gives a different prediction, and in all three cases the “Polymorphism” prediction was automatically assigned using allele frequency data; ^4^ KGGSeq filtered the variants by their maximum frequency among sub-populations nested in the database: in this case, the variant has an allele frequency of 0.08 in the East Asian population in ExAC and was therefore filtered; * Possibly representing a sequencing error due to (1) tri-allelic reads noted in manual inspection of alignment and (2) indel situating at end of a poly-(A) tract.

## Supplementary Materials

Supplementary materials can be found at www.mdpi.com/1422-0067/18/4/770/s1.

## Abbreviations

CMT	Charcot-Marie-Tooth Disease
DI-CMT	Dominant intermediate CMT
