# Supplementary material for: Rapid Identification of Pathogenic Variants in Two Cases of Charcot-Marie-Tooth Disease by Gene-Panel Sequencing
Source: Int J Mol Sci. 2017 Apr 5;18(4):770. doi: 10.3390/ijms18040770 (PMC5412354; doi:10.3390/ijms18040770)
Supplement: Supplementary file 1 [file ijms-18-00770-s001.zip › ijms-183383-suppl/Supplementary material I.pdf]

Supplementary material I

Validation of variant-calling pipeline using NIST Genome in a Bottle data

VCF	BED	Reference	SNV Recall	SNV Precision	Indel Recall	Indel Precision
[1] ILMN-GIAB.hc.raw.vcf-36719704	TruSeq Exome v1.2	NA12878 NIST Genome in a Bottle v3.2.2 (hg19)	96.57%	99.11%	89.12%	92.64%
[2] ILMN-GIAB.snp.filtered.vcf-36719704	TruSeq Exome v1.2	NA12878 NIST Genome in a Bottle v3.2.2 (hg19)	96.37%	99.45%	89.12%	92.64%
[3] ILMN-GIAB.snp_indel.filtered.vcf-36719704	TruSeq Exome v1.2	NA12878 NIST Genome in a Bottle v3.2.2 (hg19)	96.37%	99.45%	85.78%	93.41%

Validation of local variant-calling pipeline was performed using NIST Genome in a Bottle dataset provided by Illumina. Briefly, FASTQ data was downloaded from the BaseSpace website (https://basespace.illumina.com/analyses/36605989?projectId=25504495). The FASTQ files downloaded were processed as with actual clinical samples. The variants called were either passed raw [1] or after filtering by VQSR of SNPs [2] or additionally with indel filtering by VQSR [3], to the BaseSpace website for validation. Recall and percentage percentages were calculated using the Variant Calling Assessment Tool (v3.0). The values in section 4.3 of the case report represent the unfiltered, raw output from HaplotypeCaller (GATK, Broad Institute, U.S.A.) to simulate the experimental conditions.
