# Supplementary material for: Rapid Identification of Pathogenic Variants in Two Cases of Charcot-Marie-Tooth Disease by Gene-Panel Sequencing
Source: Int J Mol Sci. 2017 Apr 5;18(4):770. doi: 10.3390/ijms18040770 (PMC5412354; doi:10.3390/ijms18040770)

Supplementary material II

Manual verification of shortlisted variant calls

Case 1

1. chr1:156109095_156109095delA (likely sequencing error / miscalled variant)

LMNA:NM_170707:cDNA.2405_2405delA

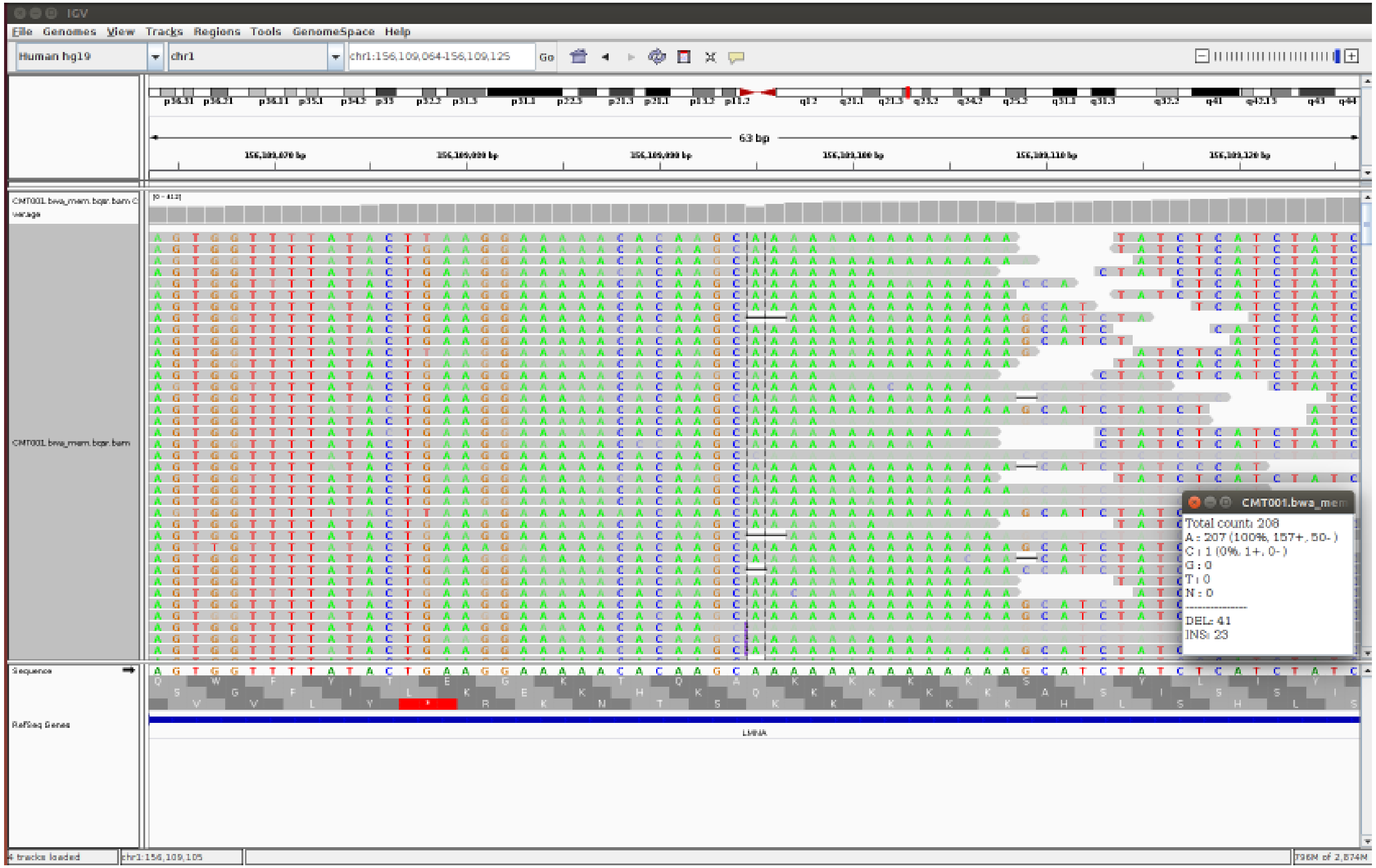

2. chr7:75932279G>A [Homozygous]

HSPB1:NM_001540:c.G250A(p.G84R)

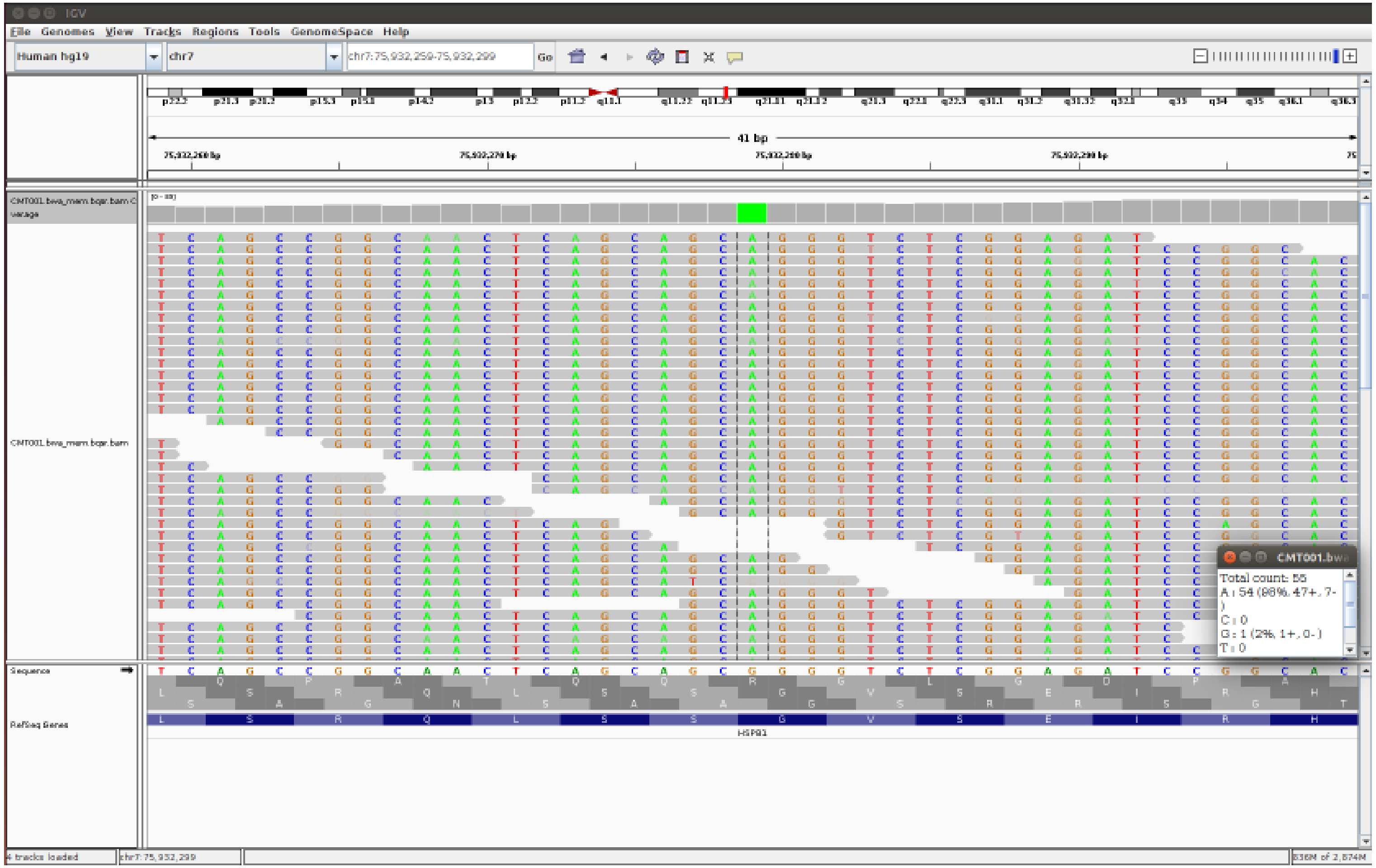

3. chr11:9861208G>C [Heterozygous]

SBF2:NM_030962:c.C3292G(p.L1098V)

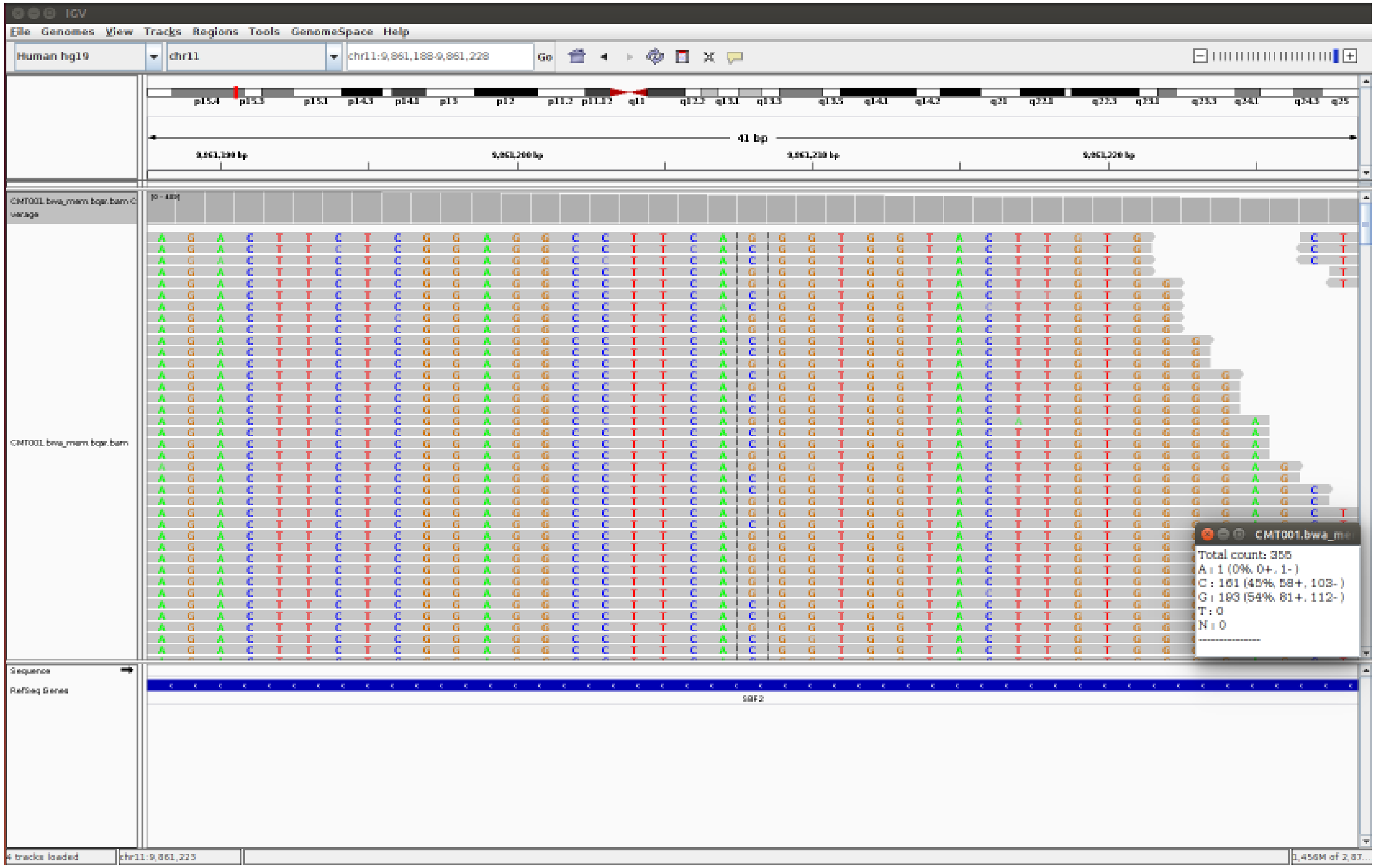

4. chr11:95595177A>G [Heterozygous]

MTMR2:NM_016156:c.T447C(p.Y149Y)

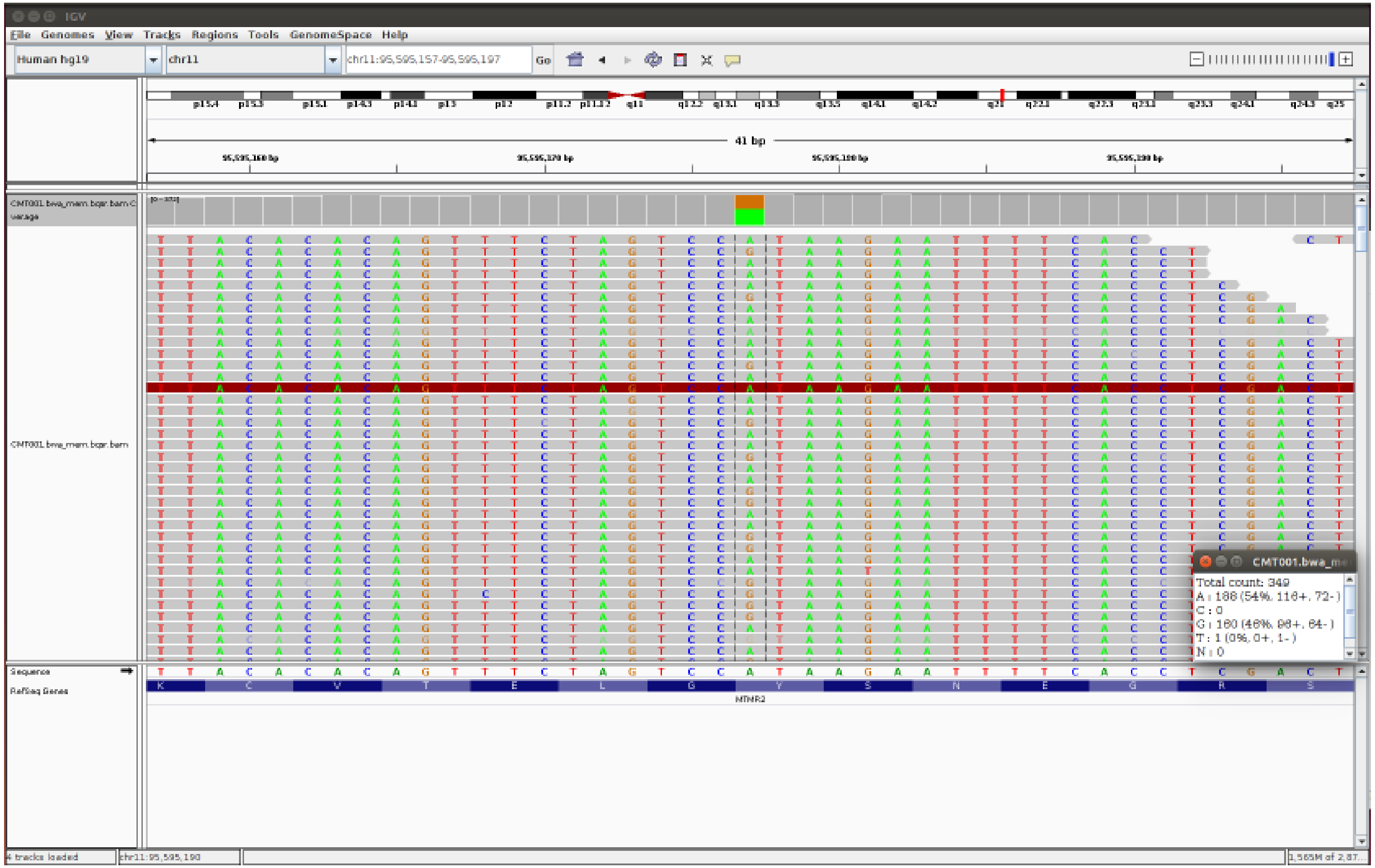

Case 2

1. chr1:10342522G>A [Heterozygous]

KIF1B:NM_015074:c.G1227A:p.T409T

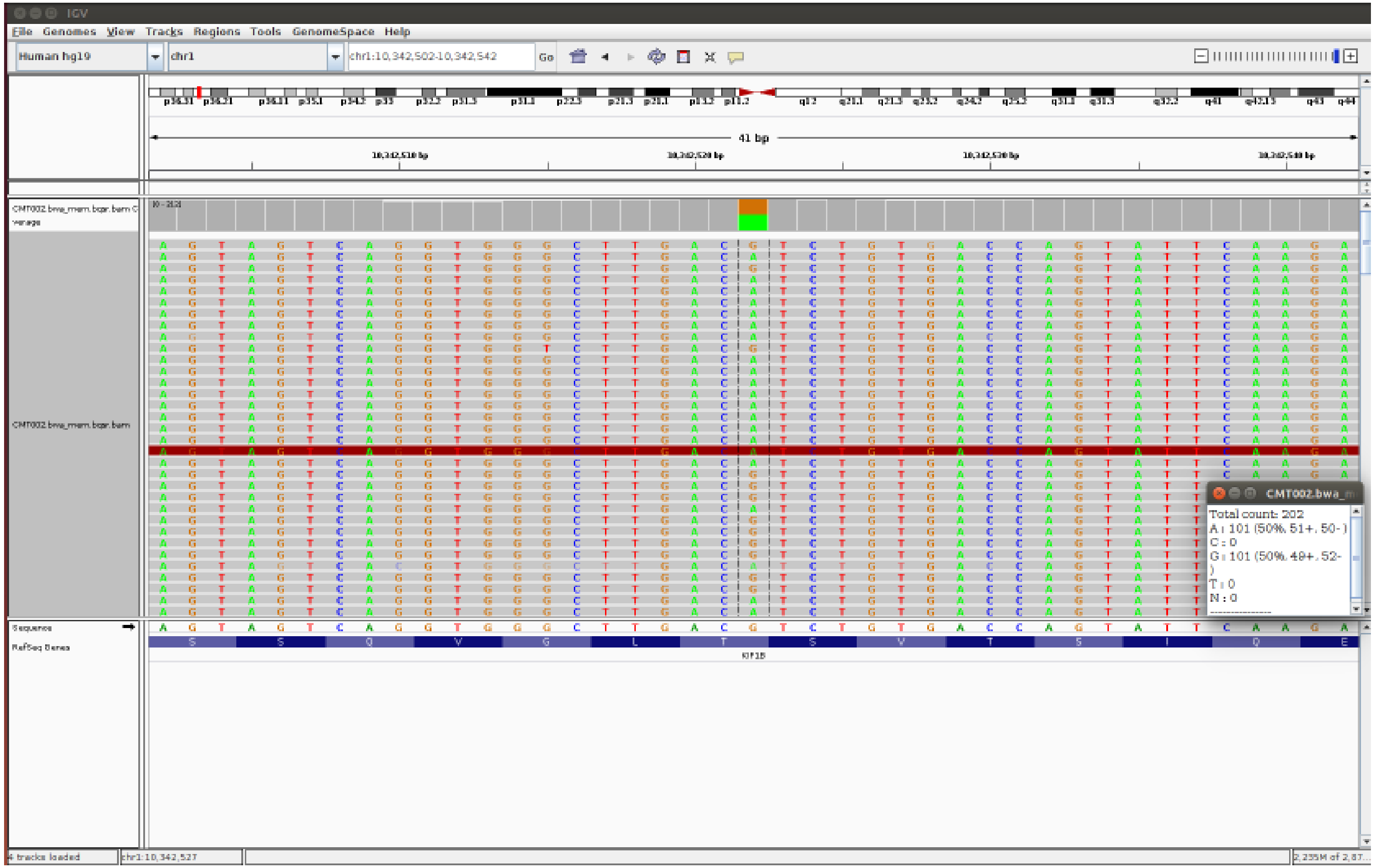

2. chr1:10397567A>G [Heterozygous]

KIF1B:NM_015074:c.A3260G:p.Y1087C

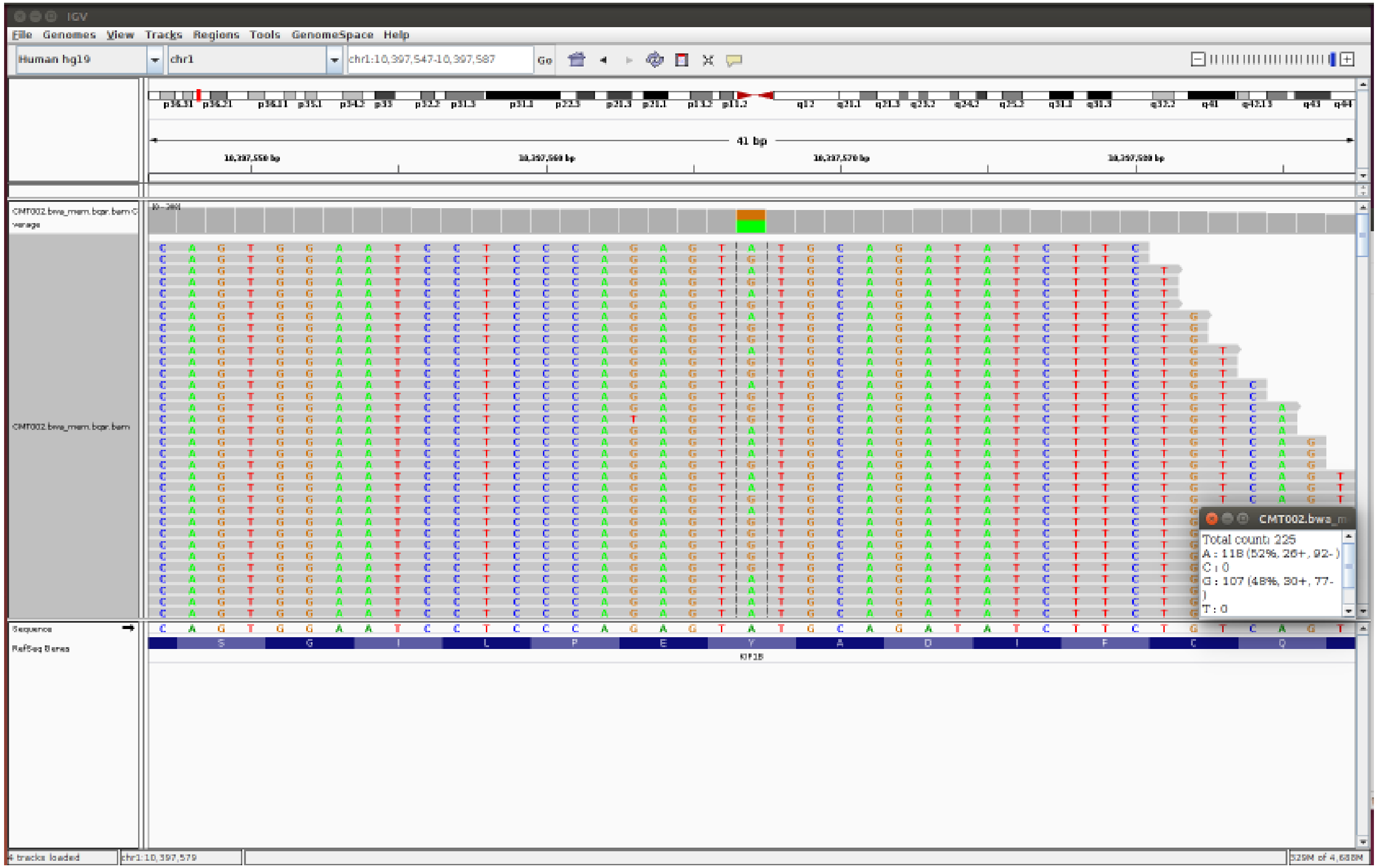

3. chr1:156109095_156109095delA (likely sequencing error / miscalled variant)
LMNA: NM_170707:cDNA.2405_2405delA

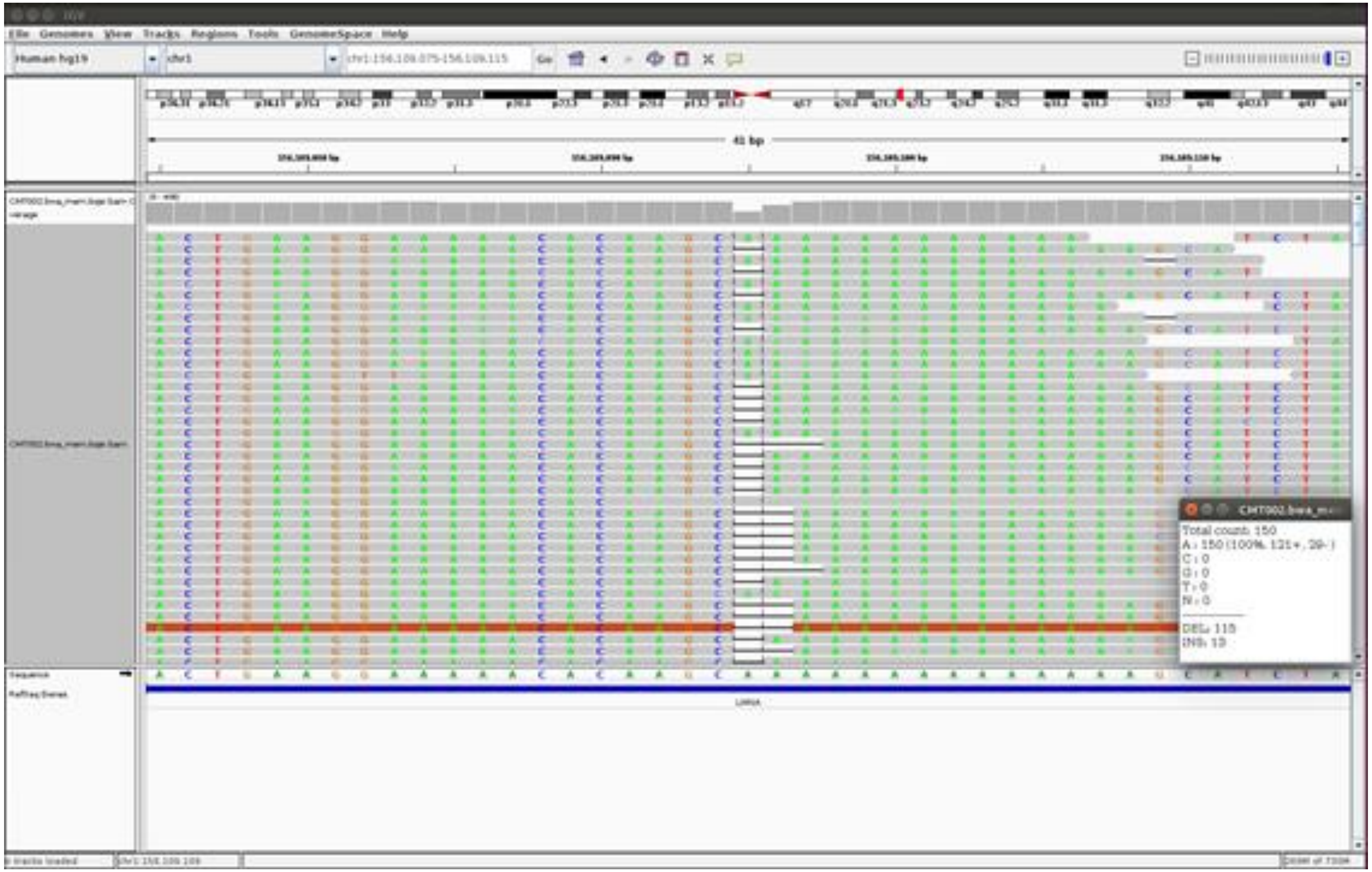

4. chr8:75272419C>T [Heterozygous]
GDAP1:NM_018972:c.C358T:p.R120W

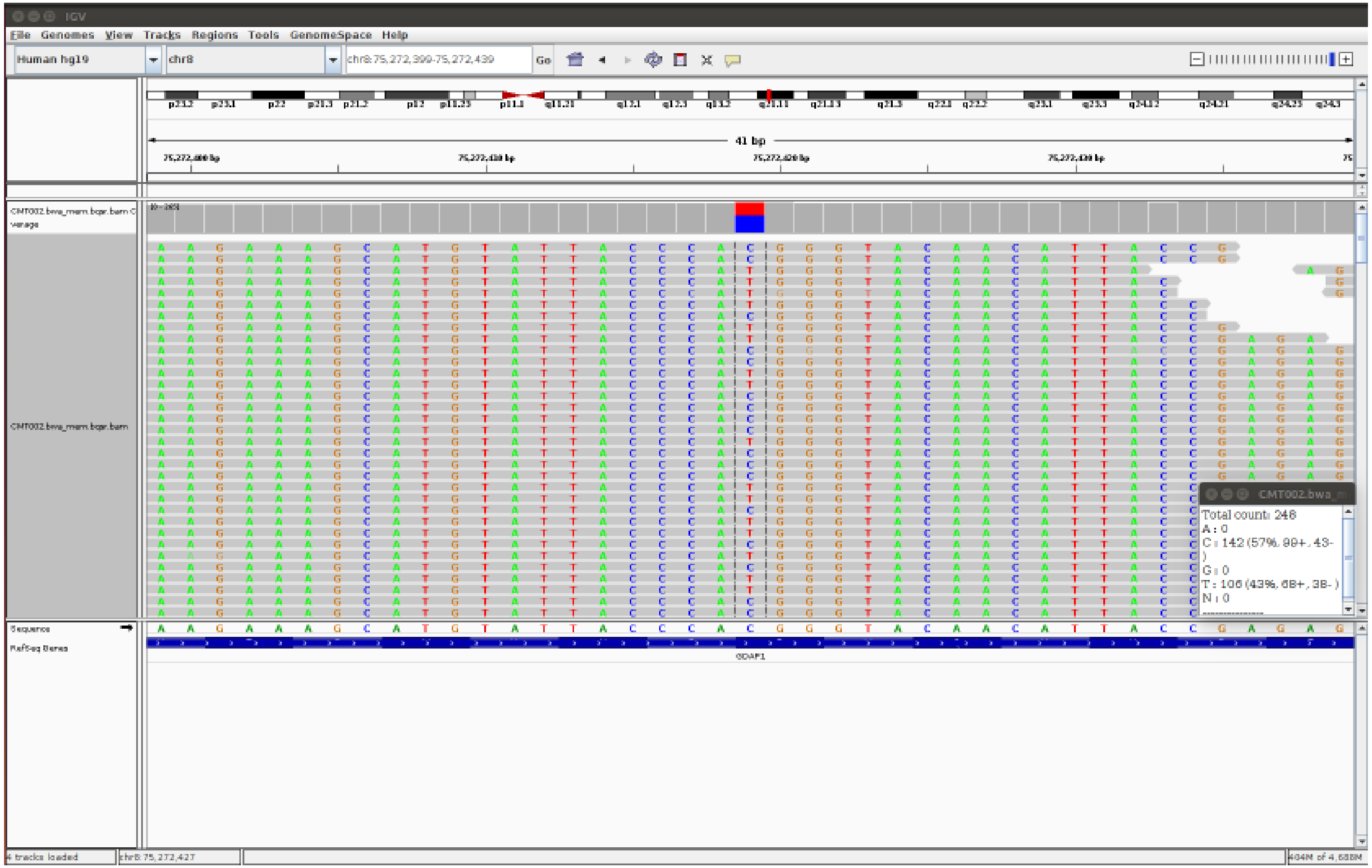

5. **chr11:9990017G>A [Heterozygous]**

SBF2:NM_030962:c.C1471T:p.L491F

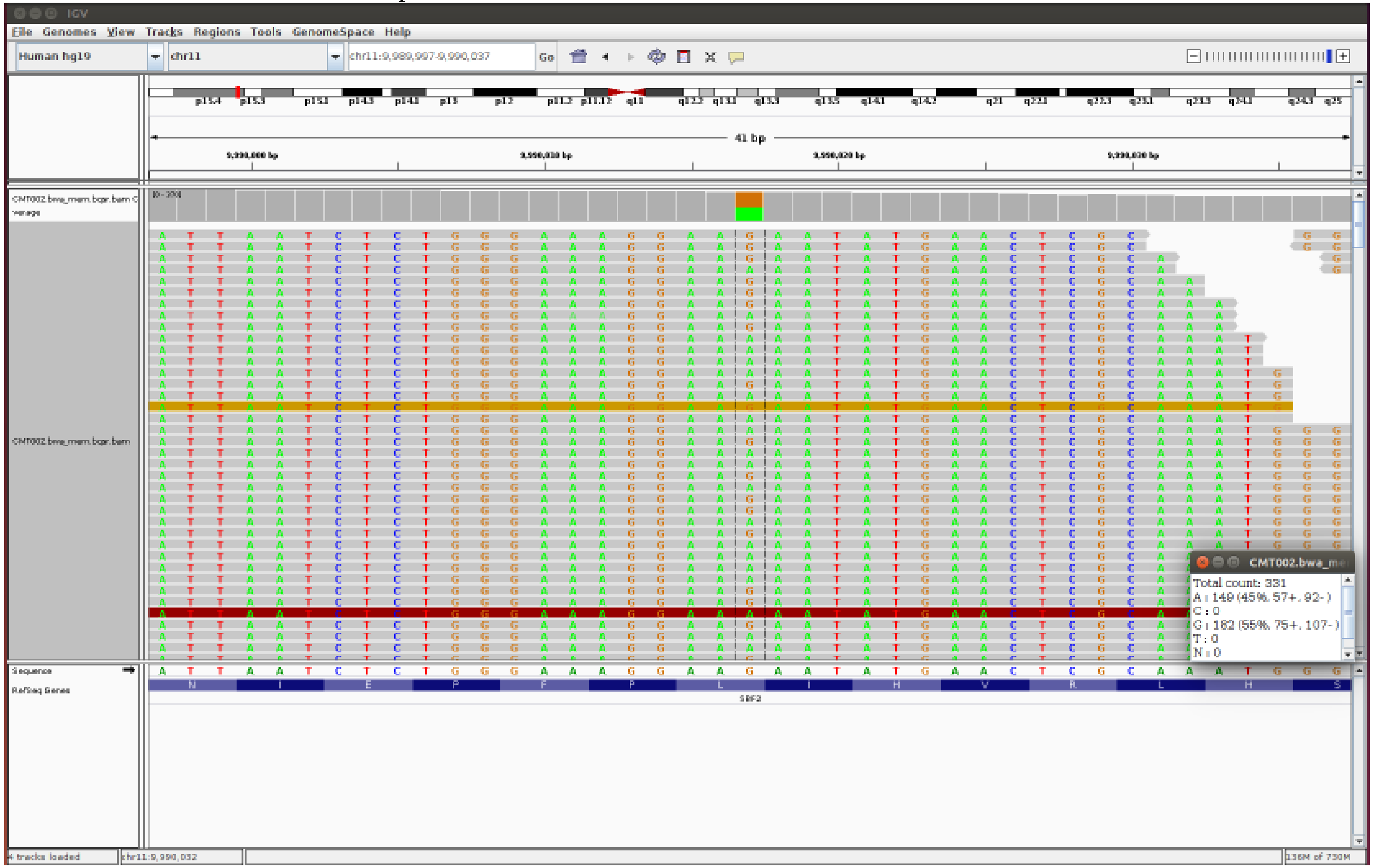

Supplement: Supplementary file 1 [file ijms-18-00770-s001.zip › ijms-183383-suppl/Supplementary material II.pdf]
